# Sick leave and work-related accidents of social workers in Germany: an analysis of routine data

**DOI:** 10.1007/s00420-018-1370-z

**Published:** 2018-10-29

**Authors:** Tanja Wirth, Dana Wendeler, Madeleine Dulon, Albert Nienhaus

**Affiliations:** 1 Competence Centre for Epidemiology and Health Services Research for Healthcare Professionals (CVcare), University Medical Centre Hamburg-Eppendorf (UKE), Martinistr. 52, 20246 Hamburg, Germany; 2 Department of Occupational Medicine, Hazardous Substances and Public Health, Institution for Statutory Accident Insurance and Prevention in the Health and Welfare Services (BGW), Hamburg, Germany

**Keywords:** Sick leave, Accident, Mental disorders, Social work, Routine data

## Abstract

**Purpose:**

The study aimed to explore the prevalence of sick leave and the risk of work-related accidents among German social workers and to describe causes and time trends in sick leave and accident claims.

**Methods:**

A retrospective analysis of routine data was carried out. Aggregated sick leave data of 195,100 social workers from four health insurance funds and 3037 accident claims of social workers from an accident insurance institution were analysed. Causes of accidents were examined by statistics of the German Social Accident Insurance (DGUV). Sick leave rates per 100 insured person-years were calculated. Relative risks (RR) of accidents were calculated in a multivariate analysis for three occupational groups (social workers and therapists, caregivers in sheltered workshops and teachers in residential institutions) and compared to other health and welfare service workers.

**Results:**

Mental disorders caused about one-fifth of the sick leave days of social workers. Sick leave due to mental disorders slightly increased in 2015 compared to 2012 (+ 3% and + 18%). Among the three subgroups of social workers, caregivers in sheltered workshops (RR 1.30; 95% CI 1.14–1.49) and teachers in residential institutions (RR 1.41; 95% CI 1.17–1.70) were at an increased risk of accidents at the workplace. Accidents were mostly caused by slipping (30%) and by violence (22%).

**Conclusions:**

This study confirms that sick leave of social workers is frequently caused by mental disorders. Future studies could further examine differences between practice fields, long-term effects of work hazards and effective workplace interventions.

**Electronic supplementary material:**

The online version of this article (10.1007/s00420-018-1370-z) contains supplementary material, which is available to authorized users.

## Introduction

The human service sector is a highly demanding practice field and known for its adverse psychosocial work environment (Rugulies et al. 2007). Among the human service professionals, social workers are one large occupational group. Social workers commonly reported an effort–reward imbalance (Rugulies et al. 2009), high emotional demands (Drüge and Schleider 2016), extensive workload, role conflicts (Blomberg et al. 2015; Tham and Meagher 2009) and role ambiguity in former studies (Coyle et al. 2005; Lloyd et al. 2002). One-fifth of social workers in Danish and German studies were exposed to physical violence (Madsen et al. 2010; Steinlin et al. 2015). In a Finish study sample, 11% of social workers experienced moral distress, which is the emotional burden of a discrepancy between professional values and actual practice (Mänttäri-van der Kuip 2016). Among different practice fields of social work, child welfare was identified as being especially demanding (Kim 2011; Tham and Meagher 2009).

Such job demands have been associated with mental disorders and sick leave among human service professionals (Borritz et al. 2005, 2010; Rugulies et al. 2007). Accordingly, human service professionals had a higher risk of a mood disorder (standardised incidence ratio (SIR) 1.11–2.11; Hannerz et al. 2009), disability pension due to mental diagnoses (Hazard Ratio (HR) 1.41; Samuelsson et al. 2013) and long-term sick leave (Odds Ratio (OR) 1.42; Aagestad et al. 2016) compared to other occupational groups. Recent studies have also supported that social workers experience high levels of stress and resulting burnout (Borritz et al. 2006; Lloyd et al. 2002; Sánchez-Moreno et al. 2015). The prevalence of burnout measured by high scores on the emotional exhaustion subscale ranged from 56% to more than 66% (Anderson 2000; Evans et al. 2005; Sánchez-Moreno et al. 2015). In a German sample of child welfare workers, 18% had a risk of burnout (Steinlin et al. 2016). In register-based studies, Wieclaw et al. (2005, 2006) found higher risks of hospitalisation for affective and stress-related disorders among social workers compared to clerical staff [Relative Risks (RR) 1.72–2.09] and non-human service professionals (HR 1.47–2.73).

Only a few studies have analysed routine data on sick leave and compensation claims among social workers. In a cohort study, Finnish and Swedish social workers were at a greater risk of work disability with mental diagnoses than preschool teachers (RR 1.43–1.77), special education teachers (RR 1.50–1.91) and psychologists (RR 1.46–1.53; Rantonen et al. 2017). Roberts et al. (2015) found that social and welfare professionals had a fourfold greater risk of developing mental injuries than managers and other professionals.

Social insurance funds in Germany hold large databases on events of sick leave and work-related accidents. To the best of our knowledge, analyses of these data have solely focused on sick leave due to back pain or cardiovascular diseases (Liebers et al. 2016) or have displayed only limited results for social workers (e.g. BBK Bundesverband 2008; Grobe and Steinmann 2015; Meyer and Meschede 2016). Work-related accidents have not been taken into account. This means that social workers’ sick leave and compensation claims from statutory health and accident insurances in Germany have not yet been analysed comprehensively. Therefore, the aim of this study was to explore the prevalence of sick leave and risk of work-related accidents among social workers using routinely collected data from German statutory health insurance funds and accident insurance institutions.

With respect to health insurance data, we asked the following research questions: (1) What are causes of sick leave among social workers? (2) Do sick leave days due to mental disorders differ by gender, age or field of social work? (3) Are there changes in diagnosis-specific sick leave days of social workers between 2012 and 2015 (time trend)? With respect to accident insurance data we asked: (4) Do social workers have higher risks of work-related accidents compared to other health and welfare service workers? (5) Are there changes in the number of accidents from 2011 to 2015 (time trend) (6) What are causes of accidents among social workers?

## Methods

A retrospective data analysis was carried out on routine data from German statutory health and accident insurances. Sick leave days of social workers in 2012 and 2015 registered by four different health insurance funds were analysed. Additionally, accident claims from 2011 to 2015 of social workers insured with the BGW were examined. Causes of accidents were examined by projected sample statistics of the DGUV from 2011 to 2015.

### Data sources

The social security system in Germany consists of five individual pillars. One of these pillars is the statutory health insurance. Statutory health insurance funds cover approximately 90% of the German population. About 113 different funds exist, which are organised in six larger associations. They protect their members against financial risks of diseases (GKV-Spitzenverband 2017). Information on sick leave of social workers was requested from five statutory health insurance funds or their associations. Four of them provided aggregated anonymised data for the year 2015 (AOK-Bundesverband, BARMER GEK, BKK Dachverband e. V. and Techniker Krankenkasse); two could additionally deliver data from 2012 for the analysis of time trends. The aggregated data were given for sex, 5-year age groups and—for three funds—also for different fields of social work. Fields of social work were classified according to the German Classification of Economic Activities 2008 (Statistisches Bundesamt 2008).

A second pillar is the statutory accident insurance. It is organised by the German Social Accident Insurance (DGUV), which is the umbrella association of 34 individual insurance institutions (DGUV 2016). One of these is the Institution for Statutory Accident Insurance and Prevention in the Health and Welfare Services (BGW), which is responsible for various non-public companies in the social work sector, such as welfare associations, preventive care and rehabilitation properties, and pedagogical establishments. Amongst others, it compensates work-related accidents of employees. A database of the BGW was available for analyses of work-related accidents; it contained all accident claims from 2011 to 2015. Besides claim data, the database contained information on gender, date of birth, profession and industry. Individual accident insurance institutions such as the BGW only record detailed information about accidents—such as causes of accidents—for a random sample of 7% of accident claims. The DGUV receives claim data from samples of all 34 accident insurance institutions—thus, having a wider database. Analyses of the DGUV were, therefore, requested for the description of causes of accidents. The DGUV provided projected sample statistics.

### Study population

Aggregated sick leave data of employed members aged between 15 and 69 years who were registered as social workers were requested from health insurance funds. Since 2012, professional activities are coded according to the German classification of occupations (KldB) of 2010 (BA 2011). For the purpose of this study, social workers were defined as those professionals registered under the four digit code 8312—occupations in social work and social pedagogics. Health insurance funds provided sick leave data of 195,100 insured person-years of social workers (fund 1: 53,581; fund 2: 49,914; fund 3: 31,831; fund 4: 59,774). Thus, aggregated data of about two-thirds of social workers registered in Germany in 2015 are presented here (Statistik der BA 2016).

Accident insurance institutions only receive information about professional activities of employees who claim an accident. Their professional activities are coded according to the International Standard Classification of Occupations in the version of the DGUV (ISCO-HV; DGUV 2009). Social work associate professionals (ISCO-HV: 346) were included in this study. They comprised the following three subgroups, which were analysed separately: 34601—social workers, social therapists and outreach workers who mainly work in support services or residential facilities (hereafter referred to as social workers and therapists), 34602—caregivers in sheltered workshops who mainly assist the disabled in their social and vocational rehabilitation, 34603—teachers in residential institutions. They mainly support and care for children, families, the elderly and the mentally ill in homes and residential facilities or work in child day-care. We hereafter refer to all three groups as social workers.

### Sick leave

In Germany, employees are obligated to present a physician’s certificate to their health insurance fund if they take sick leave lasting longer than 3 days. In this study, sick leave was defined as the number of medically certified sick leave days recorded by health insurance funds. Causes of sick leave were the diagnoses reported by physicians on the sick note to health insurance funds. These are categorised according to the International Classification of Diseases (ICD-10-GM; DIMDI 2014). Mental disorders included ICD-10 codes F00-F99. As sick leave data differ between funds, for example, some consider multiple diagnoses and others only the main diagnosis, data of different funds could not be merged.

### Accident claims

The BGW receives accident claims of employees by the employer or occupational physician of the company. Reporting is mandatory for accidents, which are either fatal or cause sick leave of employees for more than 3 days. Accidents can be differentiated by commuting accidents and accidents at the workplace. In this study, the outcome measure comprised the number of reportable accidents at the workplace. The cause of accident was defined as the last event deviating from normality which was leading directly to the accident.

### Ethical considerations and data protection

In this study, only anonymised data were used. Hence, no advice was obtained on questions of professional ethics and professional conduct from an Ethics Committee. For reasons of data protection, two health insurance funds did not display results for aggregated groups of less than 50 members or with less than 100 sick leave cases. Likewise, the DGUV did not stratify their data for small samples.

### Statistical analyses

Health insurance data were analysed descriptively. Sick leave days were standardised to 100 insured person-years and stratified by diagnosis (question 1). For reasons of comparison, sick leave days from two health insurance funds were age-standardised. Direct standardisation was carried out to the standard population of employed persons in Germany in 2010 (Statistisches Bundesamt 2011). Stratified analyses by gender, age group and field of work were performed (question 2). Time trends in sick leave between 2012 and 2015 were explored descriptively (question 3).

From BGW data absolute numbers of accident claims were derived. In a multivariate regression model, RRs with 95% confidence intervals (CI) of accidents at the workplace of social workers and therapists, caregivers in sheltered workshops and teachers in residential institutions were calculated (question 4). All other employees insured with the BGW with an accident claim in 2015 served as the reference group (other health and welfare service workers). These included, for example, hairdressers and healthcare professionals. The analysis was adjusted for age and gender. The total number of social workers insured with the BGW, or specifically of those without compensation claims, is unknown. Thus, the number of commuting accidents was used as an estimate for the calculation. For this purpose, it was expected that commuting accidents occur among social workers with the same possibility as in other health and welfare service workers. This seems plausible, as fluctuations in the numbers of commuting accidents are primarily caused by weather conditions. Time trends from 2011 to 2015 and causes of accidents were explored descriptively (question 5 and 6). All statistical analyses were performed using Excel® 2013 spreadsheets or SPSS® Statistics Version 21.

## Results

Three quarters of social workers among health insurance funds were female. The age distribution was similar across all four funds. About 17% were under the age of 30 years, 51% were between 30 and 49 years, and 32% were 50 years or older. Social workers insured with fund 2 were slightly older; 47% were above the age of 50 years. Results of accident insurance institutions are reported for the three subgroups of social workers. About 70% of social workers and therapists, 60% of caregivers in sheltered workshops and 80% of teachers in residential institutions were female. The age distribution was comparable to those of health insurance funds. Characteristics of study populations from health insurance funds, the BGW and the DGUV are displayed in the Electronic Supplementary Material (Tables S1–3).

### Sick leave

Overall, between 128 and 151 cases, and 1406 and 1773 sick leave days/100 insured person-years of social workers were reported to the four different health insurance funds in 2015. A single case of sick leave lasted on average 11–13 days (data not shown).

Across all funds, mental disorders caused most sick leave days of both female and male social workers (females: 316–431 days/100 person-years; males: 224–347 days/100 person-years). About 21% of sick leave days were thus caused by mental disorders. Respiratory diseases were the second most common cause of sick leave (about 18%), followed by musculoskeletal diseases (about 14%; Table 1).

**Table 1:** Diagnosis-specific sick leave days of social workers for four health insurance funds stratified by gender, 2015. Source: data provided by Wissenschaftliches Institut der AOK (WIdO), BARMER GEK, BKK Dachverband e. V., Techniker Krankenkasse; presentation by the author

Diagnosis (ICD-10 chapter)	Fund 1^a^		Fund 2^b^		Fund 3		Fund 4^b^	
	Days^c^	%	Days	%	Days	%	Days	%
Women								
Musculoskeletal	391	15.5	245	14.4	230	15.5	219	12.8
Mental	424	16.9	431	25.4	316	21.3	396	23.1
Respiratory	397	15.8	318	18.7	304	20.5	318	18.6
Circulatory	112	4.5	42	2.5	43	2.9	36	2.1
Digestive	114	4.5	79	4.6	61	4.1	72	4.2
Injury	166	6.6	144	8.5	108	7.3	124	7.2
Others	909	36.2	440	25.9	420	28.3	546	31.9
Men								
Musculoskeletal	262	13.2	163	13.2	189	16.1	163	12.1
Mental	347	17.4	319	25.9	224	19.1	307	22.8
Respiratory	321	16.1	231	18.8	257	21.9	268	19.9
Circulatory	132	6.6	57	4.6	48	4.1	56	4.1
Digestive	112	5.6	70	5.7	72	6.2	73	5.4
Injury	172	8.6	119	9.7	107	9.2	140	10.4
Others	644	32.4	273	22.2	275	23.5	339	25.2

^a^Data of fund 1 include multiple diagnoses

^b^Rates of fund 2 and 4 are age-standardised to the standard population of employed persons in Germany 2010 (Statistisches Bundesamt 2011)

^c^Sick leave days per 100 insured person-years

**Fig. 1:**
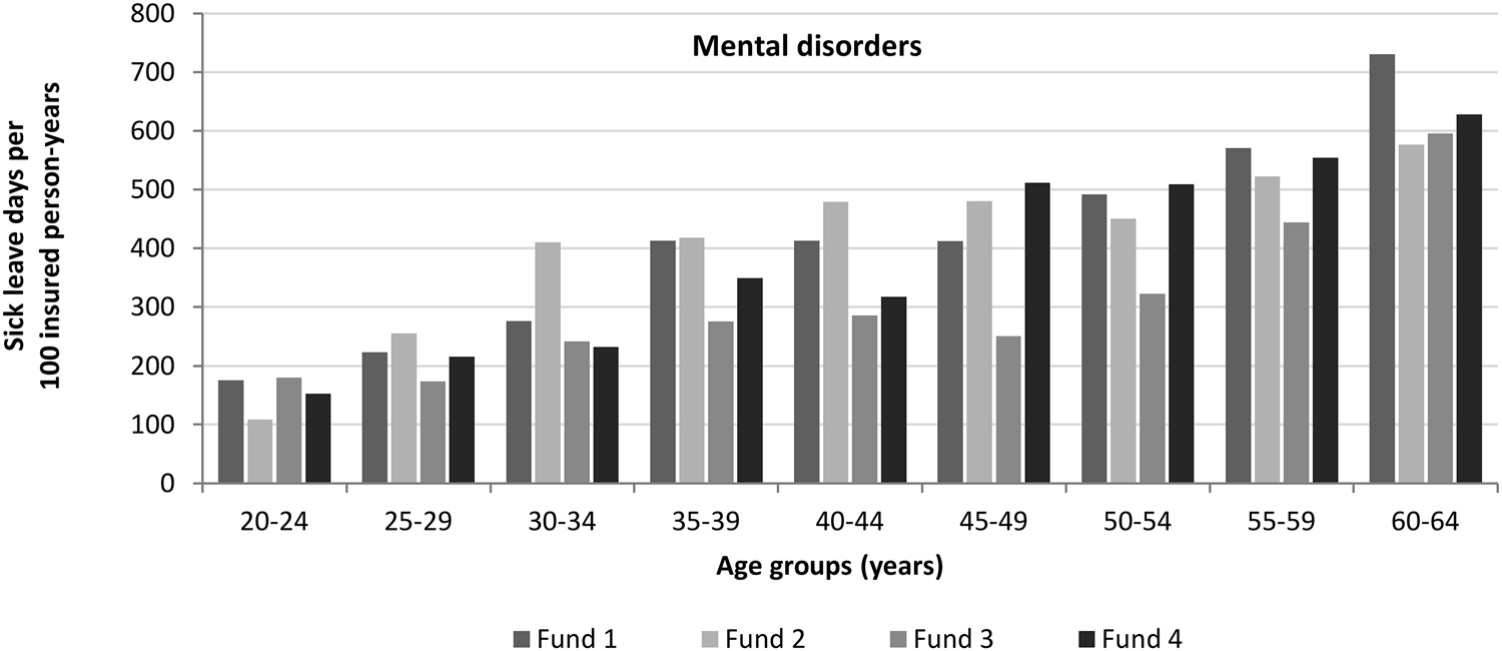
Sick leave days due to mental disorders of social workers for four health insurance funds by age group, 2015. Source: data provided by Wissenschaftliches Institut der AOK (WIdO), BARMER GEK, BKK Dachverband e. V., Techniker Krankenkasse; presentation by the author

**Fig. 2:**
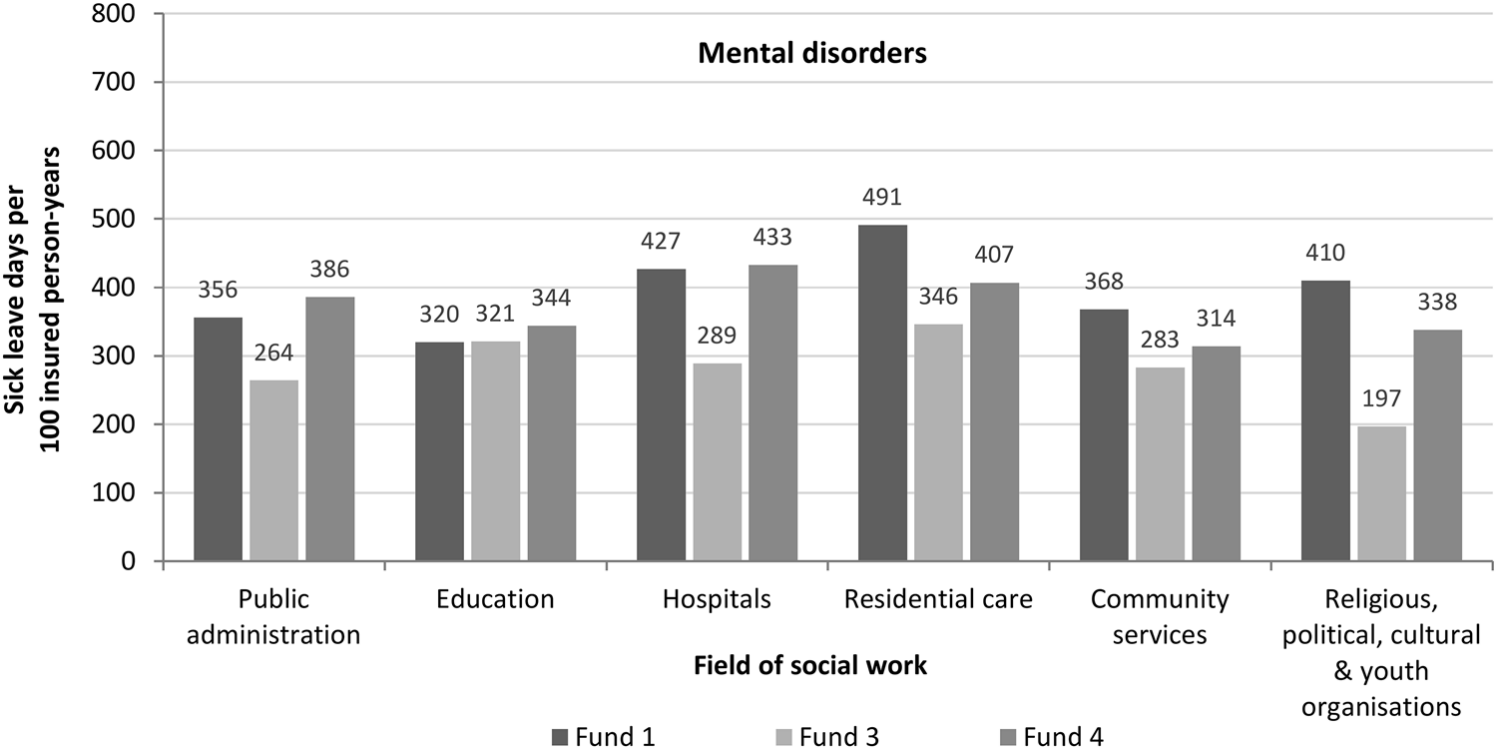
Sick leave days due to mental disorders of social workers for three health insurance funds stratified by field of social work, 2015. Notes: Residential care includes nursing homes, homes for children, the elderly and disabled and other homes for people in need; education includes pre-primary and secondary education and adult, cultural and recreation education; community services include, for example, social services for the elderly and disabled, child day-care and refugee aid. Source: data provided by Wissenschaftliches Institut der AOK (WIdO), BARMER GEK, BKK Dachverband e. V., Techniker Krankenkasse; presentation by the author

Female social workers had higher sick leave rates than their male counterparts across virtually all funds and all diagnoses (e.g. fund 1, mental disorders: 424 vs. 347/100; Table 1). The number of sick leave days due to mental disorders of social workers increased with age. Social workers aged between 20 and 24 years had a rate of less than 200 sick leave days/100 insured person years. Those at the ages of 35–39 years had a rate of about 350/100. This rate increased to 600/100 at the ages of 60–64 years (Fig. 1). Figure 2 displays annual sick leave rates due to mental disorders for the most common fields of social work. Social workers employed in residential care and hospitals had the highest rates. Two health insurance funds each registered over 400 sick leave days/100 insured person-years for these fields in 2015 (Fig. 2).

Two funds provided data for the analysis of time trends in sick leave days. In 2015 compared to 2012, sick leave rates had increased due to mental disorders (fund 1: + 3%; fund 4: + 18%) as well as due to diseases of the respiratory system (fund 1: + 21%; fund 4: + 26%). Only marginal changes were observed for other diagnoses (data not shown).

### Accidents at the workplace

In 2015, 4276 accidents (1239 commuting accidents; 3037 accidents at the workplace) were claimed by social workers insured with the BGW. Social workers and therapists had a lower risk of an accident at the workplace than other health and welfare service workers (RR 0.82; 95% CI 0.75–0.89). Conversely, an increased risk was observed for caregivers in sheltered workshops (RR 1.30; 95% CI 1.14–1.49) and teachers in residential institutions (RR 1.41; 95% CI 1.17–1.70) compared to other health and welfare service workers (Table 2).

**Table 2:** Relative Risks (RR) of accidents at the workplace among social workers compared to other health and welfare service workers, 2015

Occupation	Accidents at the workplace (*n*)	RR^a^	95% CI
Social workers			
Social workers and therapists	1619	0.82	0.75–0.89
Caregivers in sheltered workshops	945	1.30	1.14–1.49
Teachers in residential institutions	473	1.41	1.17–1.70
Other health and welfare service workers	68,916	1	

For the calculation of RRs, commuting accidents served as an estimate of the total number of social workers insured with the Institution for Statutory Accident Insurance and Prevention in the Health and Welfare Services (BGW)

^a^Adjusted for age and gender

Between 2011 and 2015, there was an overall increase of 15% in the annual number of accidents at the workplace claimed by social workers (from 2630 to 3037 accidents). The greatest increase of 40% was observed among teachers in residential institutions (from 339 to 473 accidents; data not shown).

According to analyses of the DGUV, 30% of accidents at the workplace suffered by social workers and therapists, and teachers in residential institutions were caused by slipping, stumbling or falling. Among caregivers in sheltered workshops, most accidents (22%) were caused by physical violence or frightening or surprising situations. A lower proportion of accidents of social workers and therapists (14%) and teachers in residential institutions (16%) were caused by such violent attacks (Table 3).

**Table 3:** Causes of accidents at the workplace of social workers, 2011–2015. Source: Department Statistics, German Social Accident Insurance (DGUV)

Cause of accident	Social workers and therapists (*N* = 9551)		Caregivers in sheltered workshops (*N* = 4683)		Teachers in residential institutions (*N* = 2564)	
	*n*	%	*n*	%	*n*	%
Slipping, stumbling or falling	2930	30.7	961	20.5	753	29.4
Movements of the body without physical stress	1495	15.7	961	20.5	591	23.1
Movements of the body under/with physical stress	1879	19.7	795	17.0	488	19.0
Violence, attack, threat, fright, surprise	1354	14.2	1031	22.0	410	16.0
Others/not specified	1894	19.8	935	20.0	322	12.6

As data refer to projected sample statistics, uncertainties in the extrapolation and rounding errors may occur

## Discussion

Mental disorders caused about one-fifth of the sick leave days of both female and male social workers in Germany. Thus, mental disorders were the most frequent cause of sick leave among social workers. Sick leave due to mental disorders was frequent in even young and middle-aged social workers. Furthermore, data indicate that sick leave due to mental disorders and respiratory diseases had slightly increased in 2015 compared to 2012.

The sick leave rates of social workers can be compared to the average sick leave rates of all members of health insurance funds which represent the general working population. Our age-standardised rates of mental disorders of social workers of fund 4, which holds the largest population of social workers in our study, are higher than those of the general working population reported by that fund (females: 396 vs. 345 days; males: 307 vs. 208 days; Grobe and Steinmann 2016). Similarly, Finnish and Swedish social workers had higher rates of sick leave and disability pension with mental diagnoses than preschool teachers, special education teachers and psychologists (Rantonen et al. 2017). Further studies showed higher risks of hospitalisation for affective and stress-related disorders among social workers in Denmark compared to non-human service professionals (Wieclaw et al. 2006) and clerical staff (Wieclaw et al. 2005).

The finding that female social workers had higher sick leave rates due to mental disorders than their male counterparts is in line with findings on gender differences in sick leave (Dietrich and Stengler 2013). In our study, sick leave days due to mental disorders increased with age. This corresponds with findings that older age is generally associated with longer periods of sick leave (Beemsterboer et al. 2009; Meyer 2014). However, we observed a high rate of sick leave days due to mental disorders already at the ages of 35–39 years (about 350/100 insured person-years). Our results further suggest that sick leave days due to mental disorders vary between fields of social work. Higher rates were observed in hospitals and in residential care (over 400/100 insured person-years). In former studies, child welfare was identified as a being an especially demanding practice field of social work (Kim 2011; Tham and Meagher 2009). Due to the classification system used by health insurance funds, we could not present separate results for the field of child welfare. However, the field of residential care in our study included, amongst others, homes for children and young people which are part of child welfare.

We observed an increase of over 20% in days of sick leave due to diseases of the respiratory system between 2012 and 2015 from the data of two funds, which probably has been caused by a severe wave of influenza in 2015 (Grobe and Steinmann 2016; Grobe et al. 2016; Knieps and Pfaff 2016; Meyer and Meschede 2016). A slight increase in sick leave due to mental disorders was also shown (+ 3% and + 18%), which could be a case of annual fluctuations. However, the result of an increase is in line with observations from health insurance funds in Germany across the whole working population (Grobe and Steinmann 2015; IGES Institut GmbH 2013). Similarly, the incidence of work-related mental disorders reported by occupational physicians in Britain increased from 1996 till 2009. The average increase in annual incidence was especially high for health and social care employees (+ 6%). Musculoskeletal diseases decreased over the same time period (Carder et al. 2013). The reasons for this shift in work-related diseases are still controversial. A change in occupational hazards, higher physicians’ awareness and expertise in diagnosing mental disorders, a broader acceptance among patients and increased psychological demands and stress at work are possible explanations (Carder et al. 2013; IGES Institut GmbH 2013). For the social work sector, it is observed that progressive ‘economisation’ of the sector has influenced working conditions (Banks 2011; Buestrich and Wohlfahrt 2008). Adverse working conditions such as a high workloads, role ambiguity and role conflicts are known to be associated with burnout and long-term sick leave (Borritz et al. 2005, 2010). Therefore, changing conditions such as higher workloads could also cause more work-related stress and an increase in mental disorders among social workers.

Our study showed that caregivers in sheltered workshops (RR 1.30) and teachers in residential institutions (RR 1.41) had a greater risk of accidents at the workplace compared to other health and welfare service workers. We could not observe an increased risk among social workers and therapists (RR 0.82). Reasons for these differing results in the three social work groups could lie in different work tasks or in methodological issues. For example, demographic and occupational information from routine data for these groups is sparse and we could not control for further possibly confounding variables in the analyses. A study examining workers’ compensation claims in Victoria, Australia, found that social and welfare professionals had a nearly threefold elevated risk of all injuries when compared to managers and other professionals. The risk of mental injury was increased fourfold (Roberts et al. 2015). One reason why we did not observe such high risks could be that other health and welfare service workers insured with the BGW were our control group and possibly had relatively high risks of accidents themselves.

In our study, accidents at the workplace were mostly caused by slipping or stumbling (21–31%). Among caregivers in sheltered workshops, 22% of accidents were caused by a surprising situation or physical violence. Workplace violence is a frequent problem in the care sector (Zeh et al. 2009). Our numbers only refer to accident claims and are relatively small compared to the prevalence of physical violence found in other studies. In a German survey, 51% of employees in sheltered workshops had experienced some kind of physical violence in the previous 12 months. As a consequence of violence, 15% reported a visible injury (Schablon et al. 2012). Other studies found that one-fifth of social workers were exposed to physical violence at the workplace (Madsen et al. 2010; Steinlin et al. 2015). It is likely that physical violence is underrepresented in accident claims, as these incidents might not lead to an injury reportable to accident insurance institutions.

According to the results of this study, workplace interventions for social workers should focus on their mental health. As we found high rates of sick leave due to mental disorders already at the ages of 35–39 years, interventions should already target young and middle-aged social workers. For caregivers in sheltered workshops who work with the disabled, possibilities of preventing work-related accidents due to violent incidents should be considered. However, literature on effective workplace interventions for social workers is sparse. Interventions involving elements of teaching and/or bolstering resilience showed the most promising effects in reducing compassion fatigue, which is stress resulting from exposure to traumatised individuals and can lead to serious mental disorders (Cocker and Joss 2016). A stress management intervention had good effects on the general mental health of social workers with high stress levels (Brinkborg et al. 2011).

### Strengths and limitations

To the best of our knowledge, this is the first study examining data from German statutory health and accident insurance on sick leave and accident claims of social workers. Inclusion of data of several funds enabled us to present results of a large study population and ensured high representability. As routine data were used, it can be excluded that a response or recall bias influenced the results of this study (Matteucci Gothe and Buchberger 2014).

Despite this, typical limitations of routine data need to be taken into account when interpreting the results, as the data were not primarily collected for research purposes. Routine data are coded by a variety of institutions and persons, which makes inaccuracies and misclassifications likely (Geyer and Jaunzeme 2014). Information on sociodemographic factors and possible confounding variables (e.g. psychosocial work characteristics) was limited.

A challenge we faced was to define the diverse group of social workers according to professional activity codes. We used a rather strict definition within data from health insurance funds (KldB 2010: 8312). Some social workers might also be included in other codes (e.g. 8315: occupations in social, educational and addiction counselling) and missed in this study. Within accident insurance institutions, we included social work associate professionals (ISCO-HV: 346). As health insurance funds and accident insurance institutions use different systems for coding professional activities, it was not possible to use the same definition for inclusion, which limits comparability.

Further limitations concerned sick leave data. Diseases that do not cause sick leave of more than 3 days are not subject to being reported and might be underestimated in this study. Sick leave data from the four health insurance funds could not be merged due to their different procedures, for example, in recording diagnoses (main diagnosis or multiple diagnoses) for a case of sick leave (Meyer 2014). In general, it cannot be concluded whether days of sick leave were a direct cause of hazards at work or of other factors such as personal circumstances.

In accident claim data, accidents not subject to mandatory reporting were a priori not included. Accident claims of the BGW only refer to employees of non-public institutions. However, we included analyses of the DGUV for the description of causes of accidents which also cover those of public institutions. When interpreting the risk estimates based on accident insurance data, it needs to be taken into account that the total number of social workers insured with the BGW is unknown.

## Conclusions

This study confirms that sick leave of social workers in Germany is frequently caused by mental disorders. High rates of sick leave are due to mental disorders already manifested in young and middle-aged employees. We observed some differences between fields of social work, which need further investigation. Former studies which examined a specific practice field of social work mainly focused on child welfare (e.g. Kim 2011; Tham and Meagher 2009). Thus, more research could be useful on specific demands in other fields. To reduce sick leave due to mental disorders among social workers, effective workplace interventions which combine both person-directed and work-directed preventive measures could be implemented and evaluated. Risks of work-related accidents were relatively low among social workers. Future studies could examine the long-term effects of work hazards, by including routinely collected data from the German pension office on disability retirement, which were not part of this study.

## Electronic supplementary material

Below is the link to the electronic supplementary material.

Supplementary material 1 (DOCX 15 KB)
