# Supplementary material for: Sick leave and work-related accidents of social workers in Germany: an analysis of routine data
Source: Int Arch Occup Environ Health. 2018 Oct 29;92(2):175–84. doi: 10.1007/s00420-018-1370-z (PMC6341039; doi:10.1007/s00420-018-1370-z)
Supplement: Supplementary file 1 — Supplementary material 1 (DOCX 15 KB) [file 420_2018_1370_MOESM1_ESM.docx]

Sick leave and work-related accidents of social workers in Germany – analysis of routine data

International Archives of Occupational and Environmental Health

Tanja Wirth, Dana Wendeler, Madeleine Dulon, Albert Nienhaus

**Corresponding author**

Tanja Wirth

Competence Centre for Epidemiology and Health Services Research for Healthcare Professionals (CVcare),

Institute for Health Services Research in Dermatology and Nursing (IVDP),

University Medical Centre Hamburg-Eppendorf (UKE)

Martinistr. 52

20246 Hamburg, Germany

E-mail: [t.wirth@uke.de](mailto:t.wirth@uke.de)

**Supplementary Table S1** Characteristics of social workers insured with the different health insurance funds, 2015

| **Characteristics** | **Fund 1**  (53,581  person-years) | **Fund 2**  (49,914  person-years) | **Fund 3**  (31,831  person-years) | **Fund 4**  (59,774  person-years) |
| --- | --- | --- | --- | --- |
|  | % | % | % | % |
| **Gender** | | | | |
| Female | 76.3 | 75.3 | 75.1 | 76.2 |
| Male | 23.7 | 24.7 | 24.9 | 23.8 |
| **Age (years)** | | | | |
| <30 | 17.3 | 9.9 | 17.8 | 16.0 |
| 30-39 | 28.1 | 20.4 | 25.6 | 26.1 |
| 40-49 | 22.9 | 22.7 | 25.0 | 23.9 |
| 50-59 | 24.6 | 34.5 | 25.4 | 27.5 |
| ≥60 | 7.2 | 12.4 | 6.3 | 6.5 |

Source: data provided by Wissenschaftliches Institut der AOK (WIdO), BARMER GEK, BKK Dachverband e. V., Techniker Krankenkasse; presentation by the author

**Supplementary Table S2** Characteristics of social workers with an accident at the workplace insured with the BGW, 2015

| **Characteristics** | **Social workers and therapists**  (N = 1,619) | **Caregivers in sheltered workshops**  (N = 945) | **Teachers in residential institutions**  (N = 473) | **Other health and welfare service workers**  (N = 68,916) |
| --- | --- | --- | --- | --- |
|  | % | % | % | % |
| **Gender** | | | | |
| Female | 70.2 | 58.5 | 79.5 | 72.8 |
| Male | 29.5 | 41.4 | 20.3 | 26.9 |
| Not reported | 0.3 | 0.1 | 0.2 | 0.3 |
| **Age (years)** | | | | |
| ≤30 | 20.3 | 17.1 | 27.7 | 28.1 |
| 31-40 | 19.1 | 15.9 | 19.0 | 17.0 |
| 41-50 | 25.3 | 28.8 | 19.0 | 21.8 |
| 51-60 | 28.5 | 29.8 | 28.3 | 26.4 |
| >60 | 6.6 | 8.3 | 5.9 | 6.6 |
| Not reported | 0.1 | 0.1 | / | 0.1 |

Abbreviation: BGW = Institution for Statutory Accident Insurance and Prevention in the Health and Welfare Services

**Supplementary Table S3** Characteristics of social workers with an accident at the workplace, DGUV, 2011-2015

| **Characteristics** | **Social workers and therapists**  (N = 9,551) | **Caregivers in sheltered workshops**  (N = 4,683) | **Teachers in residential institutions**  (N = 2,564) |
| --- | --- | --- | --- |
|  | % | % | % |
| **Gender** | | | |
| Female | 68.5 | 61.5 | 80.9 |
| Male | 31.0 | 38.5 | 19.1 |
| Not reported | 0.5 | / | / |
| **Age (years)** | | | |
| <30 | 18.1 | 13.6 | 18.7 |
| 30-39 | 18.5 | 14.9 | 22.6 |
| 40-49 | 26.7 | 27.4 | 21.7 |
| 50-59 | 27.5 | 35.6 | 28.7 |
| ≥60 | 9.3 | 8.5 | 8.3 |

As data refer to projected sample statistics, uncertainties in the extrapolation and rounding errors may occur.

Source: Department Statistics, German Social Accident Insurance (DGUV)
